# Amino acid geochronology of the type Cromerian of West Runton, Norfolk, UK

**DOI:** 10.1016/j.quaint.2010.06.020

**Published:** 2010-12-01

**Authors:** K.E.H. Penkman, R.C. Preece, D.H. Keen, M.J. Collins

**Affiliations:** a BioArCh, Dept of Chemistry, University of York, York YO10 5DD, United Kingdom; b BioArCh, Dept of Archaeology, The Kings Manor, University of York, York YO1 7EP, United Kingdom; c Department of Zoology, University of Cambridge, Downing Street, Cambridge CB2 3EJ, United Kingdom; d Institute of Archaeology and Antiquity, University of Birmingham, Birmingham B15 2TT, United Kingdom

## Abstract

Aminostratigraphic studies of continental deposits in the UK have hitherto relied almost exclusively on data from the aragonitic shells of non-marine molluscs for dating Pleistocene sequences. This is usually based on the d/l value of a single amino acid, d-alloisoleucine/l-isoleucine (A/I), in the total shell proteins. Two genera of freshwater gastropods (*Valvata* and *Bithynia*) are used to explore the value of using multiple amino acids from the intra-crystalline fraction, which should be more protected from the effects of diagenesis than the inter-crystalline component. Results are compared from both the aragonitic shells and opercula composed of calcite, a more stable form of calcium carbonate. In order to put the amino acid data from the West Runton Freshwater Bed into perspective, statistical analyses are used to compare them with results from the Hoxnian (MIS 11) site at Clacton-on-Sea, Essex. Twelve protein decomposition indicators revealed that the results from the shells were not as clear-cut as those from the opercula. Five indicators from the *Valvata* shell suggest that West Runton is older than Clacton (at a 95% significance level), but two actually suggested a younger age. Seven indicators show that the *Bithynia* shells from West Runton are older than congeneric shells from Clacton. In marked contrast, all 12 indicators isolated from the opercula demonstrate that West Runton is significantly older than Clacton. The data are also compared with results from Waverley Wood, an important archaeological site in the English Midlands falling within the ‘Cromerian Complex’. Contrary to earlier interpretations, the new amino acid data from *Bithynia* opercula indicate that West Runton is older than Waverley Wood, a relationship now consistent with the available biostratigraphy.

## 1 Introduction

The Cromer Forest-bed Formation (CF-bF), comprising a complex and spatially varied sequence of freshwater and marine sediments, is exposed at intervals beneath Middle Pleistocene glaciogenic deposits (tills and outwash) in the cliffs and foreshore along the North Sea coast of Norfolk and Suffolk (West, 1980; Preece and Parfitt, 2000).

The CF-bF includes the West Runton Freshwater Bed (WRFB) – the stratotype for the Cromerian interglacial stage – which is exposed at the base of the cliff at West Runton, near Cromer, Norfolk (52.941 N; 1.254 E) (West, 1980; Gibbard et al., 2010). The WRFB, averaging ca. 1.6 metres thick, and comprising organic rich silts, detritus muds, reworked silt clasts, scattered small pebbles and sand, is exposed over a length of about 250 m east of West Runton Gap (Woman Hithe). As described in other contributions to this special issue, the WRFB is rich in a wide range of fossils, including pollen and plant macrofossils (Field and Peglar, 2010), beetles (Coope, 2010), non-marine molluscs (Preece, 2010), and abundant vertebrates (Stuart and Lister, 2010; West, 1980; Stuart, 1996). The basal marls of the WRFB are Late Beestonian in age, but the overlying organic deposits (the “Upper Freshwater Bed” of Reid, 1882) are thought to have formed during the first half of the Cromerian Stage (West, 1980), although evidence presented elsewhere (Rose et al., 2008) and in this issue (Gibbard et al., 2010) suggests that sedimentation was rapid and the deposits may represent far less time. The WRFB is overlain by marine sediments that may have accumulated later in the Cromerian (West, 1980) or possibly much later (cf. Preece and Parfitt, 2000).

The Cromerian Stage was originally thought to be the temperate stage that immediately preceded the Anglian (West, 1980), the latter generally correlated with Marine Isotope Stage (MIS) 12 of the deep sea oxygen isotope record (Bowen, 1999). However, consideration of molluscan and vertebrate evidence from UK sites originally correlated with the Cromerian suggests that the situation is far more complicated and as many as five distinct temperate stages may have been conflated (Preece and Parfitt, 2000, 2008; Preece, 2001; Stuart and Lister, 2001). It has become clear that these sites are likely to cover a considerable time-span between the Brunhes–Matuyama boundary and the Anglian glaciation. The climatic changes recorded in the marine and ice core records (EPICA Community Members, 2004) show several warm and cold stages and substages within this time period (∼780–450 ka), of varying intensities and durations. It is therefore probable that the large diversity in the faunal and environmental terrestrial records from ‘Cromerian’ sites represent different parts of this record (Meijer and Preece, 1996; Turner, 1996; Preece and Parfitt, 2000, 2008; Preece, 2001; Stuart and Lister, 2001).

The evolutionary transitions in the water vole lineage have proved to be particularly important in helping to distinguish sites of different age. Early Middle Pleistocene mammal faunas in Britain have been divided into an earlier group with *Mimomys savini* and a later group with its descendent *Arvicola*. The former occurs in the WRFB and the latter at sites like Westbury-sub-Mendip, Boxgrove, Waverley Wood and Sidestrand (Preece and Parfitt, 2000, 2008; Preece et al., 2009). This evidence, together with the occurrence of the extinct freshwater molluscs *Borysthenia goldfussiana* and *Tanousia runtoniana*, suggests that the WRFB did not immediately precede the Anglian but occurred much earlier in the so-called ‘Cromerian Complex’ (Meijer and Preece, 1996; Preece and Parfitt, 2000; Preece, 2001).

Correlation of these ‘Cromerian’ temperate stages with the Dutch sequence, or indeed with the deep sea record, has proved extremely difficult. It now seems that the Dutch record in the early Middle Pleistocene is not as complete as was once thought (Meijer and Preece, 1996; Meijer and Cleveringa, in preparation). Moreover, all their records are derived from borehole data, and little molluscan or vertebrate evidence is available for comparison. Correlation and ‘age-modelling’ of ‘Cromerian’ fluvial sites is possible, based on terrace stratigraphy (cf. Bridgland and Allen, 1996; Bridgland, 2000; Rose et al., 2001; Westaway et al., 2002; Lee et al., 2004), but unfortunately the West Runton site is not part of a fluvial terrace sequence. All that can be concluded is that since the WRFB contains *M. savini* (and the extinct molluscs mentioned above) and is normally magnetized, it must fall in the early part of the Brunhes Chron.

Numerical dating would be one obvious way to link the continental and marine sequences. However, sites within the ‘Cromerian Complex’ lie beyond the limits of most dating techniques (e.g. U-series and OSL) applicable to the Middle–Late Pleistocene (Walker, 2005). Attempts have been made to derive numerical ages on vertebrate material from West Runton using Electron Spin Resonance (ESR). Direct dating by ESR on tooth enamel from the West Runton elephant itself (*Mammuthus trogontherii*), and enamel from two other species produced ages between 346 ± 55 and 460 ± 80 ka (i.e. either MIS 11 or 13). However, these were thought to be serious under-estimates, which have been affected by post-depositional processes at the site (Rink et al., 1996).

Amino acid geochronology is another technique potentially applicable at these timescales. Unlike ESR dating, this technique does not yield numerical ages unless an accurate temperature record is available, but it is able to rank sites in order of relative age. Some amino acid analyses have already been undertaken on shells from the WRFB (Bowen et al., 1989) and these are reviewed below. In the light of the recent work on the stratigraphical complexity of the ‘Cromerian Complex’, it is important to establish whether various sites of early Middle Pleistocene age can be differentiated using improved methods of amino acid dating. This is the subject of ongoing research. For now it is important to provide new data from the WRFB that will help date not only the elephant, the subject of this issue, but the type site of the Cromerian Stage itself. The WRFB contains a rich molluscan fauna (Sparks, 1980; Preece, 2001, 2010) and offers the opportunity to assess the relative values of dating different biominerals formed by the same species, as well as making comparisons between species. In order to put the data from West Runton into perspective, comparative data are presented from Waverley Wood, an important archaeological site in Warwickshire also believed to fall within the ‘Cromerian Complex’ (Shotton et al., 1993; Keen et al., 2006), and Clacton-on-Sea, a much later site of Hoxnian (MIS 11) age in Essex (Bridgland et al., 1999). Whereas different opinions have been expressed about the age of Waverley Wood and whether it is earlier (Bowen et al., 1989) or later (Preece, 2001) than West Runton, there is complete agreement that Clacton is younger and immediately post-Anglian.

## 2 Previous aminostratigraphic studies at West Runton, Waverley Wood and Clacton

Amino acid geochronology potentially has the time-range to discriminate between different stages of the ‘Cromerian Complex’. All amino acids (except glycine) can occur in two forms, called stereoisomers or enantiomers, which are non-superimposable mirror images of each other. These stereoisomers were originally named by the direction in which they rotate plane-polarised light: laevo-rotatory (l) and dextro-rotatory (d). All amino acid residues in proteins are of the l-form, but this artificial enrichment of the l-form is thermodynamically unstable, so when the organism dies or when there is no more tissue-turnover, spontaneous racemization occurs until an equilibrium of d- and l-amino acids is reached. In theory, the older the sample, the more racemization will have occurred, the higher the number of d-amino acids present, and the greater the d/l value, up to the point of equilibration. Amino acid geochronology is based upon the time-dependent increase of these degradation reactions within the protein fraction of a biomineral, such as the shells of molluscs.

Early methods of chemical separation, using Ion-Exchange Liquid Chromatography, were able to separate the enantiomers of one amino acid found in proteins, l-isoleucine (l-Ile, I), from its most stable disastereoisomer alloisoleucine (d-aile, A). By analysing the total protein content within the shells of non-marine molluscs from interglacial sites within the UK, an amino acid geochronology was developed using this time-dependent increase in A/I, enabling correlations to be made with the marine oxygen isotope stages. These pioneering works provided support for the existence of additional post-Anglian interglacials between the Hoxnian (MIS 11) and the Ipswichian (MIS 5e) (Miller et al., 1979; Bowen et al., 1989). However, both the correlation of the type site at Hoxne with MIS 9 (rather than MIS 11) and the conclusion that Waverley Wood was older than the type-Cromerian site at West Runton have proved controversial (e.g. Ashton et al., 1994, 1995; Bridgland, 1994; West and Gibbard, 1995; Preece, 2001; Schreve, 2001; McCarroll, 2002).

The results of the original A/I analyses on the shells of freshwater molluscs from West Runton, performed by Ion-Exchange Chromatography, are presented in Table 1, although it was also reported that some anomalously young ratios were obtained, but no further details have been published (Bowen et al., 1989). These results were consistent with the site being pre-Anglian, but younger than the site of Waverley Wood. The data from West Runton were used to suggest a correlation with MIS 13, whereas those from Waverley Wood were thought to indicate an MIS 15 age (Bowen et al., 1989; Bowen, 1999; Table 1).

In the light of the apparent conflicts between the amino acid and biostratigraphical data, a new series of analyses of multiple amino acids isolated from intra-crystalline biomineral fractions was undertaken. Comparison of the extent of protein degradation seen in shell material from these two Cromerian sites was supplemented by the analysis of material from the post-Anglian site at Clacton. The initial sample analysed for A/I from Clacton was a single *Corbicula fluminalis* valve, from the Natural History Museum collection, which yielded unexpectedly low values, originally correlated with the Ipswichian (Miller et al., 1979; Table 1), although equivalent ratios were subsequently correlated with MIS 7 (Bowen et al., 1989). However, two shells of the marine bivalve *Macoma balthica* also analysed from Clacton gave higher ratios (Miller et al., 1979). Five gastropod shells gave A/I values consistent with a correlation with MIS 11 (Bowen et al., 1989; Table 1). Channels of different ages are present at Clacton (Warren, 1955), but museum labels rarely specify the precise channel, which may account for the scatter of ages from this ‘site’. The material from Clacton analysed in the present study comes from samples correlated with the Lower Freshwater Bed at West Cliff, attributed to MIS 11 (Bridgland et al., 1999).

## 3 New analytical techniques employed here

A new method of amino acid analysis has been developed for geochronological purposes (Penkman, 2005; Penkman et al., 2007, 2008a), combining the isolation of an ‘intra-crystalline’ fraction of amino acids by exhaustive bleach treatment of ground shell carbonate (Sykes et al., 1995) with a new Reverse-Phase High Pressure Liquid Chromatography (RP-HPLC) method (Kaufman and Manley, 1998). This combination of techniques results in the analysis of d/l values of multiple amino acids from the chemically-protected protein within the biomineral (shell or operculum), enabling both decreased sample sizes and increased reliability. The intra-crystalline protein degrades within a ‘closed system’ during the burial history of the shell (Towe, 1980; Sykes et al., 1995), vital for the application of this technique for geochronological purposes (Collins and Riley, 2000).

This method of RP-HPLC determines the absolute and relative concentration of both l- and d-forms of amino acids. Proteins must be broken down into their constituent amino acids prior to analysis. Proteins spontaneously break down over geological time, but in order to ensure complete hydrolysis, samples are treated in strong mineral acid, which cleaves any remaining peptide bonds. RP-HPLC analysis can be conducted on samples both with and without this additional hydrolysis step. Two alternative measurements of the composition of the different stereoisomers are therefore obtained: the Free Amino Acid (FAA) fraction (amino acids which have been generated from spontaneous peptide bond hydrolysis) and the Total Hydrolysable Amino Acid (THAA) fraction, determined following laboratory hydrolysis. These two analyses reveal further aspects of protein decomposition beyond the extent of racemization of each amino acid, including the extent of decomposition of the free amino acids, both potentially useful indicators of age.

If the closed system is uncompromised then the extent of protein decomposition should only be affected by time and temperature. On this assumption, direct geochronological comparisons can be made between sites that are likely to have experienced the same integrated thermal history.

The stability of the mineral phase could have implications for the preservation of the proteinaceous material. Calcium carbonate commonly exists in two forms, calcite and aragonite. The crystal form of gastropod shells is aragonite, a polymorph of calcite with a rhombohedral crystal structure. Unlike in calcite, the carbonate ions do not lie in a single plane pointing in the same direction, but in two planes that point in opposite directions, destroying the trigonal symmetry of the calcite crystal. The metastable aragonite is converted to the more stable calcite form by inversion, dissolution and reprecipitation or replacement (Land, 1967). Changes in mineral phase could have implications for the preservation of the intra-crystalline fraction within the mollusc shell.

Therefore two types of molluscan biomineral were analysed during this study: the cross-lamellar aragonitic shell of the prosobranch aquatic gastropods *Valvata piscinalis* (Müller) and *Bithynia tentaculata* (Linnaeus) and *Bithynia troschelii* (Paasch), and the calcitic opercula of *B. tentaculata* and *B. troschelii*. The opercula of *Valvata* are not calcified and do not fossilise. The opercula of *B. tentaculata* can be distinguished from those of *B. troschelii* by their more elongate shape (cf. Meijer, 1985). *B. troschelii* is often referred to as *Bithynia inflata* (Hansén), especially in early English geological literature. *B. troschelii* is the only species of *Bithynia* to occur in the WRFB (Preece, 2001; Preece, 2010). However, analyses have shown that the differences in amino acid composition and protein decay patterns between the *B. tentaculata* and *B. troschelii* opercula are small and so the data between these two species have been directly compared (Penkman et al., 2008b). Although shells of the bivalves *Corbicula*, *Macoma* and *Pisidium* were analysed for A/I (Miller et al., 1979; Bowen et al., 1989; Table 1) no additional data are presented here on these species due to the difficulties in isolating identical structural layers.

The following comparisons have been undertaken:

1. Data from shells of *B. troschelii* vs data from the shells of *V. piscinalis* from West Runton.
2. Data from shells of *B. troschelii* vs opercula of *B. troschelii* from WRFB.
3. Statistical significance of data from shells and opercula from the post-Anglian site of Clacton and the pre-Anglian WRFB.
4. Data from shells and opercula from WRFB and Waverley Wood.
5. An evaluation of this methodology vs the earlier A/I data from shells.

Such comparisons will aid in the selection process of materials to be dated using these techniques.

### 3.1 Laboratory procedure

Amino acid analyses were undertaken on ten individual samples of *B. troschelii* opercula (NEaar 1452–1455, 1972–1975, 3317–2218) and four *B. troschelii* shells (NEaar 3629–3632) from the WRFB. Four *V. piscinalis* shells (NEaar 0898–0901) were also analysed from the WRFB. The samples were collected by John Clayden from the lower part of the WRFB, although unfortunately the exact relationship of these samples to the location of the elephant is not known. Four *V. piscinalis* shells (NEaar 1468–1471), four *B. tentaculata* shells (NEaar 1460–1463) and four *B. tentaculata* opercula (NEaar 1464–1467) were analysed from Clacton, taken from a deep piling at Trafalgar Road, with the samples coming from the lower levels of a freshwater bed, correlated with the Lower Freshwater Bed of Bridgland et al. (1999). The four *B. troschelii* shells (NEaar 1175, 3623–3628) and six opercula (NEaar 1176–1178, 2036–2037, 3319) from Waverley Wood were from Bulk 1, sampled from Channel 2 (Shotton et al., 1993).

All samples were prepared using the procedures of Penkman (2005); Penkman et al. (2008a). Each sample was powdered and bleached for 48 hours with 12% NaOCl to isolate the intra-crystalline fraction. Two subsamples were taken: one fraction was directly demineralised and the free amino acids analysed (referred to as the ‘Free Amino Acid’ fraction; FAA; ‘F’), and the second was treated with 7 M HCl under N_2_ at 110 °C for 24 h to release the peptide-bound amino acids, thus yielding the ‘Total Hydrolysable Amino Acid’ concentration (THAA; ‘H’). Samples were then dried by centrifugal evaporator and rehydrated for RP-HPLC analysis with 0.01 mM l-homo-arginine as an internal standard.

The amino acid compositions of the samples were analysed in duplicate by RP-HPLC using fluorescence detection following a modified method of Kaufman and Manley (1998). A sample of 2 μl is injected and mixed online with 2.2 μl of derivitising reagent (260 mM n-Iso-L-butyryl L-cysteine (IBLC), 170 mM o-phthaldialdehyde (OPA) in 1 M potassium borate buffer, adjusted to pH 10.4 with potassium hydroxide pellets). The amino acids are separated on a C_18_ HyperSil BDS column (5 mm × 250 mm) at 25 °C using a gradient elution of 3 solvents: sodium acetate buffer (solvent A; 23 mM sodium acetate tri-hydrate, 1.5 mM sodium azide, 1.3 μM EDTA, adjusted to pH 6.00 ± 0.01 with 10% acetic acid and sodium hydroxide), methanol (solvent C) and acetonitrile (solvent D). The l and d isomers of 10 amino acids were routinely analysed. During preparative hydrolysis both asparagine and glutamine undergo rapid irreversible deamination to aspartic acid and glutamic acid respectively (Hill, 1965). It is therefore not possible to distinguish between the acidic amino acids and their derivatives and they are reported together as Asx and Glx. The best resolved amino acids were Asx, Glx, serine (Ser), alanine (Ala) and valine (Val). Statistical analysis was performed using Minitab v.14.

## 4 Results

**Fig. 1:**
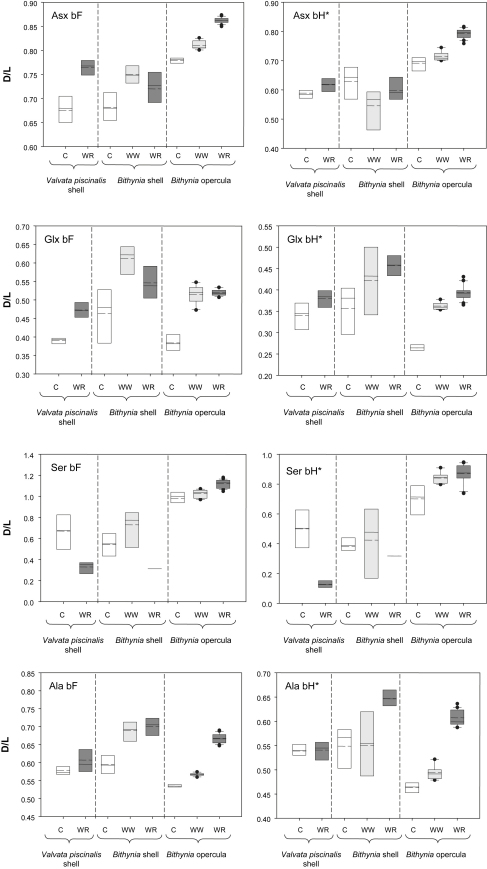

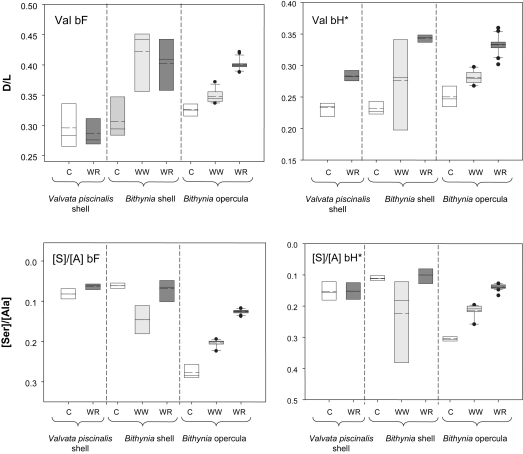
d/l values of Asx, Glx, Ser, Ala, Val and Phe, and [Ser]/[Ala] for the Free Amino Acid (FAA; bF; left) and Total Hydrolysable Amino Acid (THAA; bH*; right) fraction of bleached (intra-crystalline) *Valvata piscinalis* shells and *Bithynia* shells and opercula from Clacton (C), Waverley Wood (WW) and West Runton (WR). The species of *Bithynia* analysed from Clacton is *B. tentaculata*, but *B. troschelii* at Waverley Wood and West Runton. For each site, the base of the box indicates the 25th percentile. Within the box, the solid line plots the median and the dashed line shows the mean. The top of the box indicates the 75th percentile. Where more than 9 data points are available, the 10th and 90th percentiles can be calculated (shown by lines below and above the boxes respectively). The results of each duplicate analysis are included in order to provide a statistically significant sample size. The *y*-axes for the [Ser]/[Ala] data are plotted in reverse, so that the direction of increased protein degradation for each of the indicators remains the same. Note different scales on the *y*-axes.

The extent of racemization in five amino acids (d/l of Asx, Glx, Ser, Ala and Val), along with the ratio of the concentration of Ser to Ala ([Ser]/[Ala]), is reported for both the FAA and THAA fractions (Table 2; Fig. 1). These indicators of protein decomposition have been selected as their peaks are cleanly eluted with baseline separation and they cover a wide range of rates of reaction. It is expected that with increasing age, the extent of racemization (d/l) will increase whilst the [Ser]/[Ala] value will decrease, due to the decomposition of the unstable serine.

If the amino acids were contained within a closed system, the relationship between the FAA and the THAA fractions should be highly correlated, with non-concordance enabling the recognition of compromised samples (e.g. Preece and Penkman, 2005). One of the opercula from Waverley Wood, 2036bH*, showed this non-concordance and so was rejected from the dataset.

The ratios observed from the shells and opercula from Clacton are consistent with a MIS 11 correlation, with the data clustering with that obtained from other sites such as Swanscombe, Hoxne, Woodston, Elveden (Ashton et al., 2005) and Beeches Pit, West Stow (Preece et al., 2007). The extent of protein decomposition in the West Runton opercula is significantly higher than those from both Clacton and Waverley Wood (Fig. 1). The extent of protein decomposition in the *Valvata* and *Bithynia* shells do not show such a clear-cut distinction between the West Runton and Clacton samples, although there are several indications that the West Runton samples are more degraded and hence older.

The *Valvata* results from West Runton have the highest d/l values obtained on this species using this technique, consistent with a pre-Anglian age (Penkman, 2005). The lack of data from *Valvata* shells from older sites does not allow a maximum age to be calculated, but the protein is certainly more degraded than seen in the MIS 11 sites of Clacton, Hoxne and Woodston (Penkman, 2005). The bleached *Bithynia* shell results from the WRFB have higher d/l values than those from Clacton, with concomitant lower [Ser]/[Ala] values. These results are therefore also consistent with a pre-Anglian assignment for the WRFB.

The strength of using the multiple amino acid approach is that any geochronological interpretation is based on a suite of data. When the data are taken as a whole for both the shells and the opercula, the overall extent of protein decomposition appears to be greater in the West Runton samples than in the Clacton samples. However, the degree of difference observed in the extent of protein decomposition in the *Valvata* and *Bithynia* shells as compared to the *Bithynia* opercula in the Clacton and WRFB sites appears to be significant. An increase in the degree of relative difference between sites of different ages has important implications for the level of resolution possible. If the relative difference in protein decomposition between sites of two different ages is increased, then the level of time resolution possible is also increased, enabling a better understanding of this particularly complex time period.

The absolute values of the extent of protein decomposition at each site are different for the three materials, due to differences in the original protein composition and possibly its local environment within the mineral. A direct comparison between the levels of resolution possible in the three materials can be made by examining the relative difference between the means between the two sites, along with their 95% confidence limits. Along with the problem of small sample sizes, amino acid data has upper and lower limits (0 and 1) and therefore statistical tests must be applied with caution. However, they can provide a useful insight alongside the interpretation based on the graphical data alone. For each amino acid within each fraction (F and H) a 2-tailed *t*-test, assuming unequal variances, was performed on the Clacton and West Runton data for the *Valvata* shell, a second *t*-test for these two sites for the *Bithynia* shells and a third *t*-test for the opercula (Table 3). If the result of the *t*-test produces a *p* < 0.05, this enables discrimination between the two sites with a 95% confidence limit.

In all cases, the *p* results of the *t*-tests for the Clacton and West Runton samples for the shells are greater than or equal to that of the *Bithynia* opercula. This indicates that the use of the opercula allows a greater confidence in discriminating the age of these sites than that provided by the shell.

The data from the *Valvata* shell are not as clear-cut as those from the *Bithynia* shell or opercula. In the case of FAA Ala and Val, and THAA Asx, Ala, and [Ser]/[Ala], the means of the two sites do not show a significant difference at the 95% confidence level. Using these amino acids, the WRFB is not significantly older than Clacton. However, the data from five of the indicators (Table 3) do show that West Runton is older than Clacton. Ser, in both the FAA and the THAA fractions, has a higher degree of racemization at Clacton than at West Runton. This could indicate that Clacton is older, but the racemization kinetics of Ser are particularly complicated, as discussed below, and the errors on this dataset are large.

The data from the *Bithynia* shell allow greater confidence than for the *Valvata* in asserting that West Runton is older than Clacton, with 7 out of the 12 protein decomposition indicators leading to this conclusion. However, the THAA Asx, and FAA and THAA [Ser]/[Ala] data do not allow the discrimination of these two sites, and the concentrations of D-Ser are too low to be detectable.

In contrast, it can be stated (within 95% confidence limits) that every one of the 12 indicators of protein decomposition in the *Bithynia* opercula demonstrates more extreme protein decomposition at West Runton (Table 3). The data from the opercula therefore strongly support the hypothesis that the age of the WRFB is significantly greater than Clacton.

If discrimination between these two sites is possible with one biomineral but not the other, then the advantage of using the first can clearly be seen. However, in some cases both shells and opercula allow discrimination of the two sites in question. In that case, it is useful to quantify some measure of the degree of difference, in order to determine which biomineral provides the greatest degree of resolution.

**Fig. 2:**
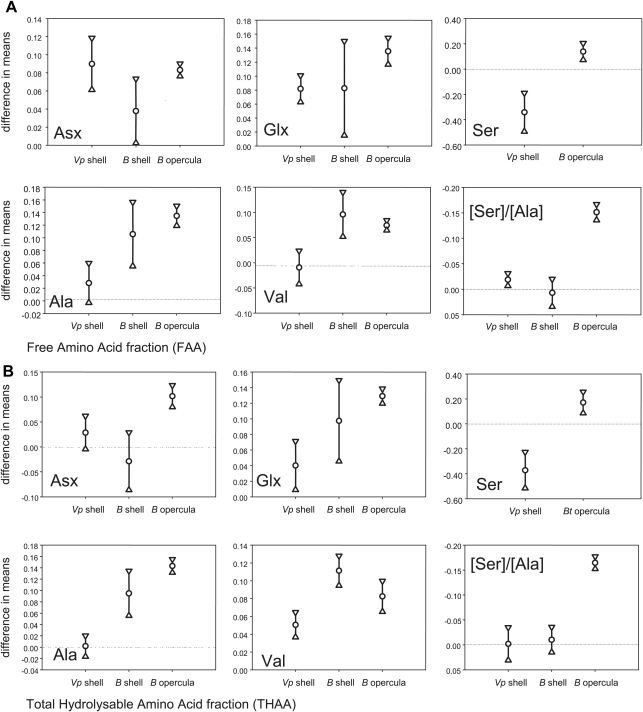
Difference in the means and 95% confidence intervals between Clacton and West Runton for racemization and decomposition in the FAA (A) and THAA (B) fractions of *Valvata piscinalis* (*Vp*) and *Bithynia* (*B*) shell and opercula. The *y*-axes for the [Ser]/[Ala] data are plotted in reverse, so that the direction of increased protein degradation for each of the indicators remains the same. The dashed line (0.00) indicates that there is no difference between the mean of the two sites. If the 95% confidence intervals fall below this zero line, then it is not possible to discriminate between Clacton and West Runton. The opercula are clearly better at discriminating between the sites than the shells, because they show a greater difference in means and have much smaller error bars. The use of opercula therefore increases the statistical confidence and temporal resolution.

The results of the 2-tailed *t*-tests allow the direct comparison of the degrees of difference between the two biominerals. The difference in the means between the two sites for each biomineral, along with the 95% confidence limits, can be compared. If the difference between the means is greater for one biomineral than the other, then the level of resolution using that biomineral is also greater. It is also important, however, to attempt to quantify the range of the data for each biomineral, which is represented by the 95% confidence limits calculated for the differences in the means. If the 95% confidence limits encompass the zero value, then no discrimination is possible at that level of confidence using that protein decomposition indicator. These results are presented graphically in Fig. 2.

The extent of racemization shows a greater degree of increase in the opercula than in the shells between each site. The measure of the extent of serine decomposition, represented by [Ser]/[Ala], which decreases with increasing age, also shows a greater decrease in the opercula samples than in the shells.

In all cases, the differences between the means for the Clacton and West Runton samples for *Valvata* shell are less than that of the *Bithynia* opercula, except in the case of Free Asx. For Free Asx, both biominerals show a large degree of difference between the two sites, but the 95% confidence limits are much smaller for the opercula dataset. Out of the 12 protein decomposition indicators, only Val (in both the FAA and THAA fraction) shows a greater difference in means between the two sites for the *Bithynia* shell as compared to the opercula. However, this greater difference is also accompanied by large errors. These results indicate that the use of the opercula allows a greater resolution in discrimination of sites of these ages than is possible using the shells.

This increase in resolution made possible by the utilisation of the opercula could therefore allow the identification of distinct events within the ‘Cromerian Complex’. *B. troschelii* shell and opercula were also analysed from Waverley Wood, in an attempt to test the relative age differences. These results (Fig. 1) again show that a higher level of discrimination is possible by using the opercula rather than the shell. The confidence limits for the *Bithynia* shell are far greater than that for the opercula. The bleached *B. troschelii* opercula results from the WRFB have higher d/l values than those from Waverley Wood, with concomitant lower [Ser]/[Ala] values. These results are therefore consistent with a pre-Anglian assignment for the WRFB, older than that represented by Waverley Wood.

## 5 Discussion

The statistical tests (Table 3, Fig. 2) show that the differences between the means of the *Bithynia* opercula from Clacton and West Runton are generally greater than the differences between the shells of *Valvata* or *Bithynia*. The individual protein decomposition indicators for the opercula are discussed below.

### 5.1 Asx

Asx is one of the fastest racemizing of the amino acids discussed here. It racemizes rapidly as a Free Amino Acid (Liardon and Ledermann, 1986; Smith and Reddy, 1989), but is unusual in that it may also undergo racemization whilst peptide bound (Brennan and Clarke, 1993) via a cyclic succinimide. For the *Bithynia* opercula the data from the WRFB show significantly greater racemization for Asx in the FAA and THAA fraction than both Clacton and Waverley Wood. It is possible to discriminate between Clacton and Waverley Wood at the 95% confidence level for the FAA fraction, although the error envelopes overlap for the THAA fraction.

### 5.2 Glx

Glx is one of the slower racemizing amino acids discussed here. The γ-carboxylate anion should activate racemization, but racemization is slowed by the formation of a lactam (pyroglutamic acid) (see Smith and Reddy, 1989; Walton, 1998). This also results in difficulties in measuring Glx in the Free form, as the lactam cannot be derivitized (i.e. labelled) and is therefore undetectable by the machine.

The samples from Waverley Wood and West Runton are all significantly higher than Clacton at the 95% confidence limit, indicating that both these sites are older. However, the Waverley Wood and West Runton values are very similar. As the rate of racemization of Glx is so slow, a large difference would not be expected in the d/l value for sites of this age. However, the d/l discriminates these ‘Cromerian’ samples from a mean Ipswichian value (∼0.2 for FAA; ∼0.15 for THAA) and from Bavel, type site of the Lower Pleistocene Bavelian stage, (∼0.8 for FAA, ∼0.6 for THAA).

### 5.3 Ser

Serine is one of the most unstable amino acids, with fast rates of racemization, in particular as a Free Amino Acid (Smith and Reddy, 1989) in which condition it also undergoes decomposition (Bada et al., 1978). These compound effects limit its usefulness for discriminating between sites at the timescales discussed in this study. The large degree of natural variability, particularly within the shell dataset, demonstrates the difficulties in interpreting the racemization data for this amino acid in samples of this antiquity.

The extent of racemization in the Free Ser in all these samples is nearing equilibrium, therefore it is impossible to discriminate between these three sites. However, the fact that the Ser has reached these high values is again important in discriminating the data from younger material.

In the THAA fraction, the measurement of the extent of racemization of Ser is complicated by its decomposition kinetics. Kinetic experiments show that the THAA fraction of Ser racemizes rapidly to a d/l ∼ 0.8 but then the ratio declines, due to decomposition of the Free Amino Acid (Bada et al., 1978), resulting in only a peptide-bound, predominately L-amino acid, fraction surviving. Interpretation of d/l Ser as an age indicator is consequently difficult, but the fact that the Ser has reached these high values is again important in discriminating the data from younger material (cf. Ipswichian values of ∼0.6).

In the FAA and the THAA fraction, the confidence intervals of all three sites overlap. However, the mean value and the lower and upper limits of the data increase in the order Clacton: Waverley Wood: West Runton.

### 5.4 Ala

Alanine (Ala) is a hydrophobic amino acid, the concentration of which is partly supplemented by the decomposition of other amino acids (most notably serine). Clacton has the lowest values in both FAA and THAA fractions, with discrimination possible at the 95% confidence limits for the samples from Waverley Wood, which have higher ratios. The samples from West Runton are clearly higher and distinguishable from those of Clacton and Waverley Wood.

### 5.5 Val

Valine has extremely low rates of racemization. The results for Val are broadly similar to that seen in the other amino acids, although with slightly less resolution possible due to the slower rates of racemization. Clacton again has lower values than the ‘Cromerian’ sites, enabling pre- and post-Anglian samples to be resolved. The extent of racemization observed at West Runton is again greater than at Waverley Wood.

### 5.6 [Ser]/[Ala]

As the protein within a sample breaks down, the concentration of Ser decreases with increasing time (5.3), and the relative concentration of Ala slowly increases. Like all other amino acids, free Ala decomposes, but it is very stable and unlike serine the rate of this reaction is extremely slow (Conway and Libby, 1958). However, it is also formed by the decomposition of more unstable amino acids, such as serine (Bada et al., 1978), resulting in a slight increase in concentration. With the isolation of a closed system of protein from molluscs (Penkman et al., 2008a,b), it is now possible to use these amino acid decomposition reactions, along with the extent of racemization, for geochronological purposes. The ratio of the concentration of Ser to Ala, [Ser]/[Ala] should decrease with increasing time in a closed system.

The [Ser]/[Ala] ratio is highest in the Clacton samples, lower in the Waverley Wood samples and lowest in the West Runton samples, enabling discrimination at the 95% confidence limits for all three sites in both FAA and THAA fractions.

### 5.7 Overall extent of protein decomposition

The conclusion drawn from the dataset is that the ‘Cromerian’ sites of Waverley Wood and West Runton show higher levels of protein decomposition at the 95% confidence level than the post-Anglian site of Clacton, using the *Bithynia* opercula. The Free Asx, Ala, Val, [Ser]/[Ala] and the Total Hydrolysable Asx, Ala, Val and [Ser]/[Ala] from the West Runton samples all lie significantly higher than that of Waverley Wood. The other reported amino acids, Glx and Ser, do not enable discrimination between sites of this age, but do allow this material to be separated from sites of significantly younger (Glx and Ser) or older (Glx) ages. With all the amino acid data taken together, the protein decomposition in the opercula at West Runton is significantly higher than that observed in opercula from Waverley Wood, indicating that West Runton is significantly older, assuming similar temperature histories. The multiple amino acid data obtained from the opercula therefore provides independent support for the molluscan and mammalian biostratigraphical interpretation (Preece and Parfitt, 2000, 2008; Preece, 2001).

There appears to be a greater degree of natural variability in the DL ratios from older sites in the bleached gastropod shell, resulting in a less clear separation between sites believed to correspond to different warm stages (Penkman, 2005). Although this could be due to reworking of the samples analysed, it is possible that the potential recrystallization of the aragonitic shell plays a significant role in the integrity of the crystal structure of the shell as samples get older. For the shells of the two species of gastropod analysed in this study, several of the amino acids did show distinct separation from the Clacton cluster, correlated with MIS 11. Despite this, the separation between the West Runton and Clacton cluster was relatively small when compared with the large separation seen with the *Bithynia* opercula. The level of resolution possible within the opercula is evidently greater than that of shells. This higher degree of resolution allows West Runton to be distinguished from other sites later in the ‘Cromerian Complex’. It has since demonstrated a significant age gap between the WRFB and the interglacial at Sidestrand, also within the CF-bF, providing useful constraints on the age of the Middle Pleistocene glacial succession in Norfolk (Preece et al., 2009).

These results from the opercula from Waverley Wood and West Runton conflict with the original A/I aminostratigraphy, which yielded ratios from West Runton younger than those at Waverley Wood (Bowen et al., 1989; Bowen, 1999). However, the data from the opercula reported here are in agreement with the prevailing molluscan and mammalian biostratigraphy (Preece and Parfitt, 2000, 2008; Preece, 2001). The factors which result in more consistent data from opercula relative to shell await further analysis. The two most obvious causes are the greater diagenetic stability of calcite and the higher level of intra-crystalline amino acids that this biomineral retains. Other studies have shown that the levels of mineral alteration of *V. piscinalis* shells within the West Runton site were undetectable by X-ray diffraction (e.g. Davies et al., 2000), but even a small degree of mineral diagenetic change, below that of the resolution of the X-ray diffraction (XRD) analysis, could affect the amino acid composition of the intra-crystalline fraction. A preliminary powder XRD study carried out here on one of the (theoretically purely aragonitic) *B. troschelii* shells from Waverley Wood shows a distinct peak at 2*θ* ∼ 29.4°, the dominant diffraction peak within a calcite spectra (Fig. 5). No calcite diffraction peak was present in the modern *Bithynia* shells analysed. Although the proportion of calcite present was not quantified, this shows clear evidence of diagenetic alteration of shell aragonite to calcite at this site. Mineral diagenesis would compromise the closed system protein within the intra-crystalline fraction; if this is a relatively common occurrence at this site/sites of this age, this would result in the greater variability and lower temporal resolution in the aragonitic shell amino acid data.

The lack of independent geochronological data for ‘Cromerian’ sites hampers the determination of an absolute age using amino acid geochronology. The temperature history of the sample affects the rate of protein breakdown and this can only be estimated if independent age estimates are available at one or more sites and all sites share a similar temperature history. Over a small geographic region, such as in this study (∼190 km; 0° 36′ latitude, 2° 42′ longitude), this latter assumption appears reasonable, but as the area of study broadens this will alter the degree of racemization in sites of equivalent ages (Wehmiller et al., 2000). However, reliable amino acid data, when integrated with alternative lines of evidence, do have the capacity to build a robust chronological framework. By comparison with data from opercula from the European continent (Penkman, in preparation), the extent of protein decomposition is consistent with an age in the early part of the Brunhes chron, most likely correlating with MIS 15 or 17.

**Fig. 3:**
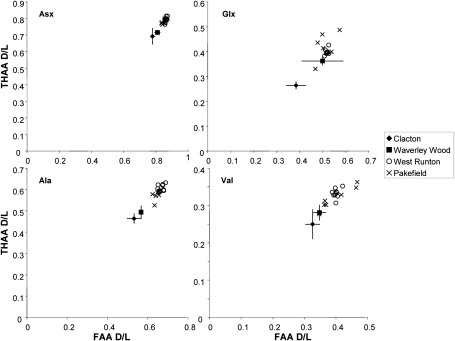
THAA vs FAA for Asx, Glx, Ala and Val d/l in the bleached (intra-crystalline) fraction of *Bithynia* opercula from Clacton, Waverley Wood, West Runton and Pakefield. Plotting the extent of racemization of THAA against FAA presents the samples in relative aminostratigraphical order based on their d/ls, with more degraded samples having higher values of d/ls for both the FAA and THAA fractions. Error bars for the Clacton and Waverley Wood data represent two standard deviations about the mean for multiple individual samples. The low concentrations from the small Pakefield opercula result in an increase in variability, particularly in Glx and Val. However, the Pakefield opercula are more racemised than those from Waverley Wood, but show similar levels of protein decomposition to samples from West Runton.

**Fig. 4:**
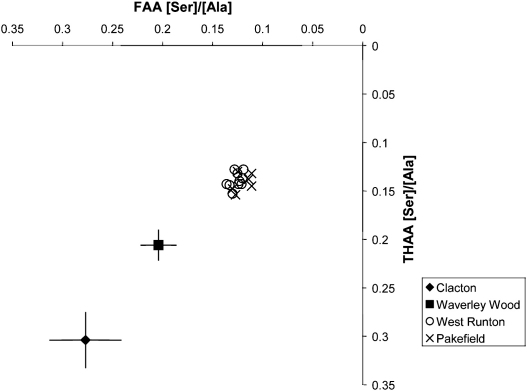
THAA vs FAA for [Ser]/[Ala] in the bleached (intra-crystalline) fraction of *Bithynia* opercula from Clacton, Waverley Wood, West Runton and Pakefield. The axes are reversed so that the direction of increased protein degradation for each of the indicators remains the same. Error bars for the Clacton and Waverley Wood data represent two standard deviations about the mean for multiple individual samples.

**Fig. 5:**
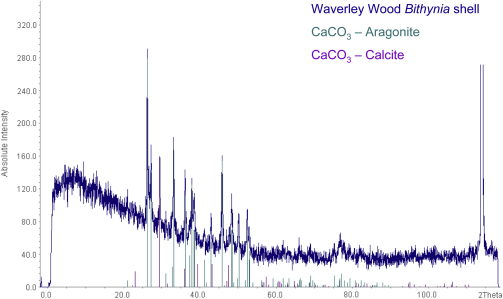
Powder X-ray diffraction analysis of a *Bithynia troschelii* shell from Waverley Wood (in blue), with reference patterns for aragonite (green) and calcite (purple); the dominant peak of each reference spectra is scaled to the intensity of the shell spectrum. *Bithynia* shells are aragonitic, and therefore this shell’s spectra should only show peaks corresponding to that of aragonite. However, a large peak in the *Bithynia* spectrum can be observed at 2*θ* = 29.4°, corresponding to the dominant diffraction peak expected in a calcite spectra. This indicates mineral diagenesis of the original aragonite to calcite in this shell, which would have compromised the closed system intra-crystalline protein.

The increase in reliability and resolution afforded by both the new methodology and substrates used in this study has enabled their use as dating evidence to push back the earliest known human occupation of Britain, and indeed of north-western Europe, at Pakefield, Suffolk (Parfitt et al., 2005). The nature of the excavations at Pakefield meant that only a few, very small opercula were recovered, resulting in greater variability than normal in the amino acid compositions due to the low concentrations. However, the Pakefield opercula have significantly higher levels of protein decomposition than that observed in samples from Waverley Wood (Figs. 3 and 4; Parfitt et al., 2005), which is a representative of a suite of sites of similar age previously thought to yield the earliest traces of human presence in northern Europe (Dennell and Roebroeks, 1996). Using the amino acids currently resolvable, the Pakefield samples show similar levels of protein decomposition to that of West Runton; they are therefore likely to be of similar age. However, it is hoped that the improvements currently being undertaken in the chromatographic methodology, increasing the number of usable amino acids, will increase the resolution and enable further differentiation of these Cromerian sites.

## 6 Conclusions

The intra-crystalline protein degradation of three materials, the shells of *Valvata* and the shells and opercula of *Bithynia*, were analysed from three sites within the UK: Clacton, Waverley Wood and West Runton. The extent of racemization of five amino acids (Asx, Glx, Ser, Ala and Val) along with a measure of the decomposition of Ser ([Ser]/[Ala]) in the FAA and THAA fractions show that the samples from the West Runton Freshwater Bed have the highest levels of protein decomposition. At the 95% confidence level, 5 and 7 out of the 12 protein decomposition indicators from the *Valvata* and *Bithynia* shells respectively show that the WRFB is significantly older than Clacton, whilst all 12 of the protein decomposition indicators from the *Bithynia* opercula support this. Statistical analysis of the difference in the means between Clacton and West Runton for each biomineral reveals that the degree of difference is greater for the *Bithynia* opercula, thereby allowing a greater level of resolution using this material. *Bithynia* opercula have been found to provide a better material for amino acid dating than shells.

The results from the *Bithynia* opercula confirm the pre-Anglian age of Waverley Wood and West Runton. The amino acids from the bleached opercula show less protein breakdown at Waverley Wood than at West Runton. These data conflict with the results of the original A/I analyses, where West Runton yielded lower ratios than Waverley Wood. However these new amino acid data do support the results from molluscan and mammalian biostratigraphy, in suggesting an age for the WRFB in the early part of the ‘Cromerian Complex’. The use of multiple amino acids and the increase in resolution afforded by the opercula allows more confidence in the assignment of WRFB to earlier than Channel 2 at Waverley Wood. This mirrors the finding that the WRFB is significantly older than the interglacial at Sidestrand within the CF-bF (Preece et al., 2009). It is too early, on the basis of the amino acid data alone, to attribute the WRFB to a particular stage in the marine oxygen isotope record, although a pre-MIS 13 age is indicated.

## Figures and Tables

**Table 1:** Amino acid data from Clacton, West Runton and Waverley Wood taken from Miller et al. (1979), Bowen et al. (1989), Bowen (1999). Note that for some samples only genera were listed in the original papers.

Site	Species	A/I	1 St dev	*n*	Original correlation	Reference
Clacton	*Corbicula fluminalis*	0.19		1	Ipswichian	Miller et al., 1979
Clacton	*Macoma balthica*	0.58	0.03	2		Miller et al., 1979
Clacton	*Pisidium*	0.305	0.001	2	MIS 11	Bowen et al., 1989
Clacton	*Valvata piscinalis*	0.299	0.002	3		Bowen et al., 1989
West Runton	*Bithynia troschelii*	0.380		1	MIS 13	Bowen et al., 1989
West Runton	*Pisidium clessini*/*amnicum*	0.346	0.031	5		Bowen et al., 1989
West Runton	*Valvata piscinalis*	0.348	0.010	9		Bowen et al., 1989
Waverley Wood	*Trichia hispida* (land snail)	0.381	0.024	3	MIS 15	Bowen et al., 1989
Waverley Wood	*Bithynia troschelii*	0.381	0.027	12		Bowen, 1999

**Table 2:** Amino acid data for West Runton, Bed D (sensu West, 1980), with data from Clacton and Waverley Wood for comparison. Error terms for the Clacton and Waverley Wood data represent two standard deviations about the mean for multiple individual samples. Error terms for the West Runton data represent two standard deviations about the mean for the duplicate analyses for an individual sample. Each sample (sh = shell; op = opercula) was bleached (b), with the Free Amino Acid fraction signified by ‘F’ and the total hydrolysable fraction by ‘H*’.

NEaar no./sample name	Species	Area	Asx d/l	Glx d/l	Ser d/l	Ala d/l	Val d/l	[Ser]/[Ala]
ClVp1-4 bF (*n* = 4)	*Valvata piscinalis* sh	Clacton, Trafalgar Road	0.675 ± 0.065	0.391 ± 0.021	0.671 ± 0.355	0.578 ± 0.033	0.296 ± 0.072	0.082 ± 0.027
ClVp1-4bH* (*n* = 4)	*Valvata piscinalis* sh	Clacton, Trafalgar Road	0.589 ± 0.073	0.340 ± 0.068	0.499 ± 0.342	0.539 ± 0.028	0.233 ± 0.030	0.154 ± 0.066
ClBt1-4bF (*n* = 4)	*Bithynia tentaculata* sh	Clacton, Trafalgar Road	0.682 ± 0.055	0.463 ± 0.147	0.543 ± 0.213	0.594 ± 0.052	0.307 ± 0.074	0.061 ± 0.013
ClBt1-4bH* (*n* = 4)	*Bithynia tentaculata* sh	Clacton, Trafalgar Road	0.629 ± 0.119	0.357 ± 0.117	0.382 ± 0.169	0.549 ± 0.091	0.232 ± 0.029	0.111 ± 0.019
ClBto1-4bF (*n* = 4)	*Bithynia tentaculata* op	Clacton, Trafalgar Road	0.779 ± 0.016	0.384 ± 0.044	0.981 ± 0.154	0.532 ± 0.036	0.326 ± 0.023	0.277 ± 0.036
ClBto1-4bH* (*n* = 4)	*Bithynia tentaculata* op	Clacton, Trafalgar Road	0.692 ± 0.050	0.264 ± 0.016	0.701 ± 0.201	0.464 ± 0.024	0.250 ± 0.040	0.304 ± 0.029
WWBtr1-4bF (*n* = 4)	*Bithynia troschelii* sh	Waverley Wood, Bulk 1	0.750 ± 0.037	0.612 ± 0.080	0.729 ± 0.388	0.690 ± 0.060	0.422 ± 0.140	0.146 ± 0.069
WWBtr1-4bH* (*n* = 4)	*Bithynia troschelii* sh	Waverley Wood, Bulk 1	0.546 ± 0.146	0.423 ± 0.161	0.424 ± 0.448	0.550 ± 0.141	0.276 ± 0.145	0.223 ± 0.286
WWBtro1-6bF (*n* = 6)	*Bithynia troschelii* op	Waverley Wood, Bulk 1	0.810 ± 0.019	0.499 ± 0.091	1.024 ± 0.076	0.567 ± 0.008	0.348 ± 0.021	0.204 ± 0.018
WWBtro1-3,5-6bH* (*n* = 5)	*Bithynia troschelii* op	Waverley Wood, Bulk 1	0.715 ± 0.008	0.362 ± 0.018	0.841 ± 0.079	0.494 ± 0.030	0.281 ± 0.021	0.206 ± 0.016
0898bF WRVp1bF	*Valvata piscinalis* sh	West Runton, Bed D	0.767 ± 0.038	0.453 ± 0.004	0.313 ± 0.094	0.654 ± 0.042	0.321 ± 0.009	0.064 ± 0.017
0898bH* WRVp1bH*	*Valvata piscinalis* sh	West Runton, Bed D	0.647 ± 0.007	0.404 ± 0.005	0.158 ± 0.018	0.553 ± 0.029	0.297 ± 0.009	0.119 ± 0.008
0899bF WRVp2bF	*Valvata piscinalis* sh	West Runton, Bed D	0.778 ± 0.007	0.494 ± 0.001	0.255 ± 0.011	0.580 ± 0.026	0.285 ± 0.026	0.073 ± 0.002
0899bH* WRVp2bH*	*Valvata piscinalis* sh	West Runton, Bed D	0.612 ± 0.009	0.383 ± 0.005	0.105 ± 0.001	0.512 ± 0.003	0.273 ± 0.015	0.169 ± 0.003
0900bF WRVp3bF	*Valvata piscinalis* sh	West Runton, Bed D	0.767 ± 0.009	0.491 ± 0.014	0.366 ± 0.023	0.578 ± 0.025	0.271 ± 0.012	0.061 ± 0.005
0900bH* WRVp3bH*	*Valvata piscinalis* sh	West Runton, Bed D	0.624 ± 0.000	0.386 ± 0.006	0.147 ± 0.017	0.543 ± 0.013	0.282 ± 0.018	0.136 ± 0.005
0901bF WRVp4bF	*Valvata piscinalis* sh	West Runton, Bed D	0.745 ± 0.005	0.453 ± 0.008	0.384 ± 0.056	0.616 ± 0.040	0.271 ± 0.015	0.055 ± 0.004
0901bH* WRVp4bH*	*Valvata piscinalis* sh	West Runton, Bed D	0.588 ± 0.001	0.350 ± 0.004	0.104 ± 0.021	0.555 ± 0.012	0.281 ± 0.015	0.184 ± 0.007
3629bF WRBtr1bF	*Bithynia troschelii* sh	West Runton, Bed D	0.731 ± 0.082	0.506 ± 0.010	0.312 ± 0.349	0.705 ± 0.033	0.351 ± 0.019	0.083 ± 0.052
3629bH* WRBtr1bH*	*Bithynia troschelii* sh	West Runton, Bed D	0.606 ± 0.114	0.426 ± 0.006	0.317 ± 0.063	0.640 ± 0.010	0.346 ± 0.010	0.099 ± 0.003
3630bF WRBtr2bF	*Bithynia troschelii* sh	West Runton, Bed D	0.750 ± 0.033	0.566 ± 0.096	ND	0.691 ± 0.288	0.410	0.109
3630bH* WRBtr2bH*	*Bithynia troschelii* sh	West Runton, Bed D	0.649 ± 0.035	0.480 ± 0.014	ND	0.651 ± 0.060	0.341 ± 0.017	0.058
3631bF WRBtr3bF	*Bithynia troschelii* sh	West Runton, Bed D	0.671 ± 0.047	0.519 ± 0.070	ND	0.681 ± 0.035	0.426 ± 0.085	0.049 ± 0.004
3631bH* WRBtr3bH*	*Bithynia troschelii* sh	West Runton, Bed D	0.550 ± 0.059	0.452 ± 0.013	ND	0.650 ± 0.002	0.330 ± 0.035	0.104 ± 0.068
3632bF WRBtr4bF	*Bithynia troschelii* sh	West Runton, Bed D	0.728 ± 0.006	0.593 ± 0.078	ND	0.724 ± 0.001	0.427 ± 0.043	0.052 ± 0.059
3632bH* WRBtr4bH*	*Bithynia troschelii* sh	West Runton, Bed D	0.591 ± 0.007	0.471 ± 0.033	ND	0.647 ± 0.065	0.358 ± 0.030	0.119 ± 0.050
1452bF WRBtro1bF	*Bithynia troschelii* op	West Runton Bed D	0.858 ± 0.013	0.520 ± 0.014	1.127 ± 0.074	0.657 ± 0.004	0.397 ± 0.003	0.133 ± 0.007
1452bH* WRBtro1bH*	*Bithynia troschelii* op	West Runton Bed D	0.774 ± 0.003	0.396 ± 0.011	0.861 ± 0.005	0.594 ± 0.017	0.329 ± 0.006	0.144 ± 0.001
1453bF WRBtro2bF	*Bithynia troschelii* op	West Runton Bed D	0.862 ± 0.011	0.521 ± 0.005	1.090 ± 0.067	0.661 ± 0.004	0.405 ± 0.013	0.120 ± 0.007
1453bH* WRBtro2bH*	*Bithynia troschelii* op	West Runton Bed D	0.798 ± 0.002	0.398 ± 0.001	0.842 ± 0.009	0.590 ± 0.008	0.325 ± 0.011	0.137 ± 0.007
1454bF WRBtro3bF	*Bithynia troschelii* op	West Runton Bed D	0.856 ± 0.016	0.508 ± 0.004	1.069 ± 0.007	0.661 ± 0.000	0.400 ± 0.04	0.130 ± 0.001
1454bH* WRBtro3bH*	*Bithynia troschelii* op	West Runton Bed D	0.764 ± 0.015	0.382 ± 0.004	0.838 ± 0.004	0.594 ± 0.009	0.307 ± 0.014	0.153 ± 0.036
1455bF WRBtro4bF	*Bithynia troschelii* op	West Runton Bed D	0.856 ± 0.008	0.511 ± 0.005	1.095 ± 0.054	0.650 ± 0.008	0.398 ± 0.000	0.121 ± 0.007
1455bH* WRBtro4bH*	*Bithynia troschelii* op	West Runton Bed D	0.790 ± 0.047	0.407 ± 0.023	0.837 ± 0.022	0.604 ± 0.018	0.347 ± 0.037	0.143 ± 0.011
1972bF WRBtro6bF	*Bithynia troschelii* op	West Runton Bed D	0.870 ± 0.003	0.526 ± 0.018	1.150 ± 0.026	0.651 ± 0.003	0.399 ± 0.007	0.124 ± 0.005
1972bH* WRBtro6bH*	*Bithynia troschelii* op	West Runton Bed D	0.792 ± 0.006	0.426 ± 0.013	0.739 ± 0.005	0.622 ± 0.003	0.332 ± 0.002	0.143 ± 0.001
1973bF WRBtro7bF	*Bithynia troschelii* op	West Runton Bed D	0.858 ± 0.002	0.527 ± 0.015	1.151 ± 0.013	0.679 ± 0.007	0.401 ± 0.003	0.123 ± 0.002
1973bH* WRBtro7bH*	*Bithynia troschelii* op	West Runton Bed D	0.797 ± 0.004	0.392 ± 0.000	0.934 ± 0.002	0.598 ± 0.000	0.337 ± 0.004	0.140 ± 0.002
1974bF WRBtro8bF	*Bithynia troschelii* op	West Runton Bed D	0.866 ± 0.002	0.517 ± 0.001	1.135 ± 0.013	0.680 ± 0.002	0.402 ± 0.003	0.119 ± 0.003
1974bH* WRBtro8bH*	*Bithynia troschelii* op	West Runton Bed D	0.797 ± 0.002	0.397 ± 0.004	0.888 ± 0.007	0.595 ± 0.008	0.335 ± 0.002	0.128 ± 0.001
1975bF WRBtro9bF	*Bithynia troschelii* op	West Runton Bed D	0.863 ± 0.001	0.516 ± 0.016	1.059 ± 0.031	0.675 ± 0.002	0.393 ± 0.001	0.125 ± 0.001
1975bH* WRBtro9bH*	*Bithynia troschelii* op	West Runton Bed D	0.792 ± 0.001	0.392 ± 0.002	0.924 ± 0.003	0.624 ± 0.000	0.328 ± 0.001	0.132 ± 0.002
3317bF WRBtro10bF	*Bithynia troschelii* op	West Runton Bed D	0.872 ± 0.006	ND	1.173 ± 0.018	0.674 ± 0.011	0.388 ± 0.001	0.136 ± 0.005
3317bH* WRBtro10bH*	*Bithynia troschelii* op	West Runton Bed D	0.814 ± 0.000	0.367 ± 0.006	0.946 ± 0.004	0.620 ± 0.021	0.336 ± 0.006	0.143 ± 0.002
3318bF WRBtro11bF	*Bithynia troschelii* op	West Runton Bed D	0.864 ± 0.003	ND	1.116 ± 0.028	0.689 ± 0.002	0.421 ± 0.003	0.128 ± 0.001
3318bH* WRBtro11bH*	*Bithynia troschelii* op	West Runton Bed D	0.814 ± 0.008	0.378 ± 0.002	0.908 ± 0.004	0.632 ± 0.011	0.352 ± 0.007	0.128 ± 0.001

**Table 3:** *p*-values from the results of the statistical 2-tailed *t*-tests, performed on amino acid data from Free Amino Acid (FAA) fraction and Total Hydrolysable Amino Acid (THAA) fraction from the shells of *Valvata piscinalis* and *Bithynia*, and opercula of *Bithynia* from Clacton and West Runton. If *p* < 0.05 (shown in bold) it is possible to distinguish between Clacton and West Runton at the 95% confidence level. ↑ indicates that the West Runton samples are more degraded than Clacton samples; ↓ indicates that they are less degraded; = indicates that they cannot be discriminated. ND = not determined. Note that whereas only 12 of the 24 measures for protein degradation of the shells indicate that West Runton is older than Clacton (with two even suggesting a younger age), in the opercula data all 12 (of the 12) measures demonstrate this.

		Asx	Glx	Ser	Ala	Val	[Ser]/[Ala]
*Valvata piscinalis*	FAA (*n* = 8)	**0.000** ↑	**0.000** ↑	**0.001** ↓	0.069 =	0.554 =	**0.006** ↑
	THAA (*n* = 8)	0.087 =	**0.013** ↑	**0.001** ↓	0.832 =	**0.000** ↑	0.903 =

*Bithynia* shell	FAA (*n* = 8)	**0.038** ↑	**0.021** ↑	ND	**0.001** ↑	**0.000** ↑	0.555 =
	THAA (*n* = 8)	0.301 =	**0.003** ↑	ND	**0.000** ↑	**0.000** ↑	0.360 =

*Bithynia* opercula	FAA (*n* = 18)	**0.000** ↑	**0.000** ↑	**0.001** ↑	**0.000** ↑	**0.000** ↑	**0.000** ↑
	THAA	**0.000** ↑	**0.000** ↑	**0.001** ↑	**0.000** ↑	**0.000** ↑	**0.000** ↑

## References

[bib1] Ashton N., Bowen D.Q., Holman J.A., Hunt C.O., Irving B.G., Kemp R.A., Lewis S.G., McNabb J., Parfitt S., Seddon M.B. (1994). Excavations at the Lower Palaeolithic site at East Farm, Barnham, Suffolk, 1989–92. Journal of Geological Society of London.

[bib2] Ashton N.M., Bowen D.Q., Lewis S. (1995). Reply to West and Gibbard. Journal of Geological Society of London.

[bib3] Ashton N.M., Lewis S.G., Parfitt S., Candy I., Keen D.H., Kemp R., Penkman K., Thomas G.N., Whittaker J.E. (2005). Excavations at the Lower Palaeolithic site at Elveden, Suffolk, UK. Proceedings of the Prehistoric Society.

[bib4] Bada J.L., Shou M.-Y., Man E.H., Schroeder R.A. (1978). Decomposition of hydroxy amino acids in foraminiferal tests: kinetics, mechanism and geochronological implications. Earth and Planetary Science Letters.

[bib5] Bowen D.Q., Hughes S., Sykes G.A., Miller G.H. (1989). Land-sea correlations in the Pleistocene based on isoleucine epimerization in non-marine mollusks. Nature.

[bib6] Bowen, D.Q., 1999. A Revised Correlation of Quaternary Deposits in the British Isles. Geological Society Special Report 23.

[bib7] Brennan T.V., Clarke S. (1993). Spontaneous degradation of polypeptides at aspartyl and asparaginyl residues – effects of the solvent dielectric. Protein Science.

[bib8] Bridgland D.R. (1994). Quaternary of the Thames.

[bib9] Bridgland D.R. (2000). River terrace systems in north-west Europe: an archive of environmental change, uplift and early human occupation. Quaternary Science Reviews.

[bib10] Bridgland D.R., Allen P., Turner C. (1996). A revised model for terrace formation and its significance for the early Middle Pleistocene terrace aggradations of north-east Essex, England. The Early Middle Pleistocene in Europe.

[bib11] Bridgland D.R., Field M.H., Holmes J.A., McNabb J., Preece R.C., Selby I., Wymer J.J., Boreham S., Irving B.G., Parfitt S.A., Stuart A.J. (1999). Middle Pleistocene interglacial Thames–Medway deposits at Clacton-on-Sea, England: reconsideration of the biostratigraphical and environmental context of the type Clactonian Palaeolithic industry. Quaternary Science Reviews.

[bib12] Collins M.J., Riley M.S., Goodfriend G.A., Collins M.J., Fogel M.L., Macko S.A., Wehmiller J.F. (2000). Amino acid racemization in biominerals, the impact of protein degradation and loss. Perspectives in Amino Acid and Protein Geochemistry.

[bib13] Conway D., Libby W.F. (1958). The measurement of very slow reaction rates; decarboxylation of alanine. Journal of American Chemical Society.

[bib14] Coope G.R. (2010). Coleoptera from the Cromerian typesite at West Runton, Norfolk, England. Quaternary International.

[bib15] Davies S.M., Rose J., Branch N.P., Candy I., Lewis S.G., Whiteman C.A., Preece R.C. (2000). West Runton (TG 188432 and TG 185432). Pre-glacial gravels, freshwater muds and coastal sand and gravels. The Quaternary of Norfolk and Suffolk. Quaternary Research Association Field Guide.

[bib16] Dennell R., Roebroeks W. (1996). The earliest colonization of Europe: the short chronology revisited. Antiquity.

[bib17] EPICA Community Members (2004). Eight glacial cycles from an Antarctic ice core. Nature.

[bib18] Field M.H., Peglar S.M. (2010). A palaeobotanical investigation of the sediments from the West Runton Mammoth site. Quaternary International.

[bib19] Gibbard P., Boreham S., Andrews J.E., Maher B.A. (2010). Sedimentation, geochemistry and palaeomagnetism of the West Runton Freshwater Bed, Norfolk, England. Quaternary International.

[bib20] Hill R.L. (1965). Hydrolysis of proteins. Advances in Protein Chemistry.

[bib21] Kaufman D.S., Manley W.F. (1998). A new procedure for determining DL amino acid ratios in fossils using reverse phase liquid chromatography. Quaternary Science Reviews.

[bib22] Keen D.H., Hardaker T., Land A.T.O. (2006). A Lower Palaeolithic industry from the Cromerian (MIS 13) Baginton Formation of Waverley Wood and Wood Farm Pits, Bubbenhall, Warwickshire, UK. Journal of Quaternary Science.

[bib23] Land L.S. (1967). Diagenesis of skeletal carbonates. Journal of Sedimentary Petrology.

[bib24] Lee J.R., Rose J., Hamblin R.J.O., Moorlock B.S.P. (2004). Dating the earliest lowland glaciation of eastern England: a pre-MIS 12 early Middle Pleistocene Happisburgh glaciation. Quaternary Science Reviews.

[bib25] Liardon R., Ledermann S. (1986). Racemization kinetics of free and protein-bound amino-acids under moderate alkaline treatment. Journal of Agricultural and Food Chemistry.

[bib26] McCarroll D. (2002). Amino-acid geochronology and the British Pleistocene: secure stratigraphical framework or a case of circular reasoning?. Journal of Quaternary Science.

[bib27] Meijer T. (1985). The pre-Weichselian non-marine molluscan fauna from Maastricht-Belvédère (Southern Limburg, The Netherlands). Mededelingen Rijks Geologische Dienst.

[bib28] Meijer T., Preece R.C., Turner C. (1996). Malacological evidence relating to the stratigraphical position of the Cromerian. The Early Middle Pleistocene in Europe.

[bib63] Meijer, T., Cleveringa, P. The Cromerian Complex Superstage in the Netherlands, in preparation.

[bib29] Miller G.H., Hollin J.T., Andrews J.T. (1979). Aminostratigraphy of UK Pleistocene deposits. Nature.

[bib30] Parfitt S.A., Barendregt R.W., Breda M., Candy I., Collins M.J., Coope G.R., Durbidge P., Field M.H., Lee J.R., Lister A.M., Mutch R., Penkman K.E.H., Preece R.C., Rose J., Stringer C.B., Symmons R., Whittaker J.E., Wymer J.J., Stuart A.J. (2005). The earliest record of human activity in northern Europe.. Nature.

[bib31] Penkman, K.E.H., 2005. Amino acid geochronology: a closed system approach to test and refine the UK model. Unpublished PhD thesis, University of Newcastle.

[bib32] Penkman K.E.H., Preece R.C., Keen D.H., Maddy D., Schreve D.C., Collins M. (2007). Testing the aminostratigraphy of fluvial archives: the evidence from intra-crystalline proteins within freshwater shells. Quaternary Science Reviews.

[bib33] Penkman K.E.H., Kaufman D.S., Maddy D., Collins M.J. (2008). Closed-system behaviour of the intra-crystalline fraction of amino acids in mollusc shells. Quaternary Geochronology.

[bib34] Penkman, K.E.H., Keen, D.H., Preece, R.C., Collins, M.J., 2008b. British Aggregates: An improved chronology using amino acid racemization and degradation of intra-crystalline amino acids (IcPD). English Heritage Research Department Report Series 6/2008.

[bib64] Penkman, K.E.H., Preece, R.C., Keen, D.H., Meijer, T., Collins, M.J. A chronological framework for British Quaternary deposits based on calcitic Bithynia opercula, in preparation. 10.1038/nature10305 PMC3162487 21804567

[bib35] Preece R.C. (2001). Molluscan evidence for differentiation of interglacials within the ‘Cromerian Complex’. Quaternary Science Reviews.

[bib36] Preece R.C. (2010). The molluscan fauna of the Cromerian type site at West Runton, Norfolk. Quaternary International.

[bib37] Preece R.C., Parfitt S.A., Lewis S.G., Whiteman C.A., Preece R.C. (2000). The Cromer Forest-bed Formation: new thoughts on an old problem. The Quaternary of Norfolk and Suffolk. Quaternary Research Association Field Guide.

[bib38] Preece R.C., Penkman K.E.H. (2005). New faunal analyses and amino acid dating of the Lower Palaeolithic site at East Farm, Barnham, Suffolk. Proceedings of the Geologists’ Association.

[bib39] Preece R.C., Parfitt S.A., Candy I., Lee J.R., Harrison A.M. (2008). The Cromer Forest-bed Formation: some recent developments relating to early human occupation and lowland glaciation. The Quaternary of Northern East Anglia, Field Guide.

[bib40] Preece R.C., Parfitt S.A., Bridgland D.R., Lewis S.G., Rowe P.J., Atkinson T.C., Candy I., Debenham N.C., Penkman K.E.H., Rhodes E.J., Schwenninger J.-L., Griffiths H.I., Whittaker J.E., Gleed-Owen C. (2007). Terrestrial environments during MIS 11: evidence from the Palaeolithic site at West Stow, Suffolk, UK. Quaternary Science Reviews.

[bib41] Preece R.C., Parfitt S.A., Coope G.R., Penkman K.E.H., Ponel P., Whittaker J.E. (2009). Biostratigraphic and aminostratigraphic constraints on the age of the Middle Pleistocene glacial succession in North Norfolk, UK. Journal of Quaternary Science.

[bib42] Reid C. (1882). The geology of the country around Cromer. Memoirs of the Geological Survey of England and Wales.

[bib43] Rink W.J., Schwarcz H.P., Stuart A.J., Lister A.M., Marseglias E., Brennan B.J. (1996). ESR dating of the type Cromerian Freshwater Bed at West Runton, U.K.. Quaternary Science Reviews (Quaternary Geochronology).

[bib44] Rose J., Moorlock B.S.P., Hamblin R.J.O. (2001). Pre-Anglian fluvial and coastal deposits in Eastern England: lithostratigraphy and palaeoenvironments. Quaternary International.

[bib45] Rose J., Juby C., Bullen M., Davies S., Branch N., Gammage Z., Candy I., Palmer A., Andy I., Lee J.R., Harrison A.M. (2008). The stratigraphy, sedimentology, palaeoenvironments and duration of the early Middle Pleistocene sediments at West Runton, north Norfolk. The Quaternary of Northern East Anglia Field Guide.

[bib46] Schreve D.C. (2001). Differentiation of the British late Middle Pleistocene interglacials: the evidence from mammalian biostratigraphy. Quaternary Science Reviews.

[bib47] Shotton F.W., Keen D.H., Coope G.R., Currant A.P., Gibbard P.L., Aalto M., Peglar S.M., Robinson J.E. (1993). The Middle Pleistocene deposits of Waverley Wood Pit, Warwickshire, England. Journal of Quaternary Science.

[bib48] Smith G.G., Reddy G.V. (1989). Effect of the side-chain on the racemization of amino-acids in aqueous-solution. Journal of Organic Chemistry.

[bib49] Sparks B.W., West R.G. (1980). Land and freshwater Mollusca of the West Runton Freshwater Bed. The Pre-glacial Pleistocene of the Norfolk and Suffolk Coasts.

[bib50] Stuart A.J., Turner C. (1996). Vertebrate faunas from the Early Middle Pleistocene of East Anglia. The Early Middle Pleistocene in Europe.

[bib51] Stuart A.J., Lister A.M. (2001). The mammalian faunas of Pakefield/Kessingland and Corton, Suffolk, UK: evidence for a new temperate episode in the British early Middle Pleistocene. Quaternary Science Reviews.

[bib52] Stuart A.J., Lister A.M. (2010). The West Runton Freshwater Bed and the West Runton mammoth: summary and conclusions. Quaternary International.

[bib53] Sykes G.A., Collins M.J., Walton D.I. (1995). The significance of a geochemically isolated intracrystalline organic fraction within biominerals. Organic Geochemistry.

[bib54] Towe K.M., Hare P.E., Hoering T.C., King K. (1980). Preserved organic ultrastructure: an unreliable indicator for Paleozoic amino acid biogeochemistry. Biogeochemistry of Amino Acids.

[bib55] Turner C. (1996). The Early Middle Pleistocene in Europe.

[bib56] Walker M.J.C. (2005). Quaternary Dating Methods.

[bib57] Walton D.I. (1998). Degradation of intracrystalline proteins and amino acids in fossil brachiopods. Organic Geochemistry.

[bib58] Warren S.H. (1955). The Clacton (Essex) channel deposits. Quarterly Journal of the Geological Society of London.

[bib59] Wehmiller J.F., Stecher H.A., York L.A., Friedman I., Goodfriend G.A., Collins M.J., Fogel M.L., Macko S.A., Wehmiller J.F. (2000). The thermal environment of fossils: effective ground temperatures at aminostratigraphic sites on the U.S. coastal plain. Perspectives in Amino Acid and Protein Geochemistry.

[bib60] West R.G. (1980). The Pre-glacial Pleistocene of the Norfolk and Suffolk Coasts.

[bib61] West R.G., Gibbard P.L. (1995). Discussion on excavations at the Lower Palaeolithic site at East Farm, Barnham, Suffolk, 1989–1992. Journal of the Geological Society of London.

[bib62] Westaway R., Maddy D., Bridgland D.R. (2002). Flow in the lower continental crust as a mechanism for the Quaternary uplift of south-east England: constraints from the Thames terrace record. Quaternary Science Reviews.

